# A bibliometric analysis of gene editing and amyotrophic lateral sclerosis (from 2004 to 2024)

**DOI:** 10.3389/fnins.2024.1499025

**Published:** 2024-11-26

**Authors:** Hejia Wan, Wenli Qian, Bingqi Wei, Kaiyue Tian, Ziyi Chen, Jiong Zhang, Fang Chen

**Affiliations:** ^1^School of Medicine, Henan University of Chinese Medicine, Zhengzhou, China; ^2^School of Nursing, Henan University of Chinese Medicine, Zhengzhou, China; ^3^School of Humanities and Social Sciences, Chinese Academy of Medical Sciences & Peking Union Medical College, Peking, China; ^4^School of Chinese Medicine, Henan University of Chinese Medicine, Zhengzhou, China

**Keywords:** amyotrophic lateral sclerosis, gene editing technology, bibliometric study, CiteSpace, VOSviewer

## Abstract

**Objective:**

To learn more about gene editing and ALS, and to provide a comprehensive view of gene editing for further treatment of amyotrophic lateral sclerosis.

**Methods:**

We searched 1981 records from Web of Science core collection and Pubmed, Scopus, of which 1,292 records were obtained after exclusion. We then scientifically and metrologically analyzed these records for spatial and temporal distribution, author distribution, subject categories, subject distribution, references, and keywords using R, software CiteSpace and VOSviewer.

**Results:**

Our analysis provides basic information about research in the field, suggests that the field has stabilized over the past decade, and identifies potential partners for interested researchers. Current research in this area is focused on inflammatory mechanisms, immune mechanisms, related diseases, and associated cytokines in ALS.

**Conclusion:**

RNA Editing, Antisense Bligonucleotide, and Glycine Receptor are cutting-edge research topics in this field, which is undergoing rapid development. We hope that this work will provide new ideas for advancing the scientific research and clinical application of ALS.

## 1 Introduction

Amyotrophic lateral sclerosis, also known as motor neuron disease, is a devastating neurodegenerative disease characterized by the degeneration of motor neurons in the brain and spinal cord, which can lead to progressive paralysis and respiratory failure. In 97% of patients, amyotrophic lateral sclerosis (ALS) is a protein disease; however, due to the heterogeneity of its pathology, ALS caused by genetic mutations is not considered a protein disease (Mead et al., 2023; Al-Chalabi and Brown, 2017). Moreover, the incidence of ALS as a disease occurs globally, with a prevalence of approximately 0.6–3.8 per 100,000 person-years, but varies from country to country. In Europe, the incidence rate ranges from 2.1 to 3.8 cases per 100,000 person-years; in Asia, the average incidence rate is 0.38 cases per 100,000 person-years in China (Gao et al., 2021a,b) and 1.2 cases per 100,000 person-years in Korea (Jun et al., 2019). In contrast, the incidence of ALS in the United States is 1.5 cases per 100,000 person-years (Berry et al., 2023). Comparatively, the incidence rate in Europe is somewhat higher (Longinetti and Fang, 2019).

However, ALS still lacks an effective treatment. Riluzole and edaravone prolong survival in ALS patients (Fang et al., 2018) and have been approved by the U.S. Food and Drug Administration for the treatment of ALS (Al-Chalabi and Brown, 2017); however, these drugs have shown very limited improvement in survival. Both drugs target nonspecific factors because of the lack of therapeutic targets and the difficulty in reaching both the brain and spinal cord. With the increasing research on disease mechanisms and therapies, gene therapies are gradually being applied to clinical design, such as central nervous system (CNS) targeting, gene delivery, gene editing, and gene knockout technologies. Targeted gene therapies for ALS include antisense oligonucleotides, RNA interference, or antibody-based approaches (Amado and Davidson, 2021). Gene editing can change the organism from the level of genes, and compared with drug therapy, if it can be applied to treatment, the theory is deep in nature and opens up new possibilities for the treatment of ALS.

Bibliometrics, as a branch of informatics, is an effective and convenient method, which takes the literature system and bibliometric characteristics as the object of study, and analyzes the literature qualitatively and quantitatively through mathematical and statistical methods (Du et al., 2022). It is now widely used in various disciplines (Wei et al., 2022), and the bibliometric data generated by using VOSviewer and CiteSpace can help to intuitively display the hotspots of research and evolution process (Ma et al., 2021). Data mining and mapping of literature in the field of gene editing in ALS can enable the allocation of medical resources and decision-making, and its contour distribution map and relationship map can help understand the quality of current research in this field, and objectively evaluate the contribution of research scholars, research countries and regions, and research institutions in this field, so that readers can intuitively understand the current status quo of the research and recognize the frontier and trend of the research (Li et al., 2023).

In recent years, this type of literature has been widely used in various fields, but there are few studies on visualizing the combination of ALS and gene editing To have a deeper understanding of the research on ALS gene editing, this study took the literature included in the Web of Science Core Collection (WoS) as the research object, and used VOSviewer and CiteSpace to analyze the literature in terms of cited articles, cited authors, keyword contributions, cited journals, and emergent keywords to analyze and map the data, and to discuss the research hotspots and research trends of gene editing in ALS therapy, with a view to provide more effective diagnostic and treatment plans for patients with ALS and provide references for future medical development.

## 2 Methods

### 2.1 Data collection

Web of Science is the leading research platform for information in the sciences, social sciences, arts and humanities, and an independent global citation database from the world’s most trusted publisher (Wei et al., 2022). Pubmed is widely browsed in the field of medicine, and is one of the most commonly used databases by medical practitioners. Scopus is a database created by Elsevier. is a database created by Elsevier, which contains many high-quality documents from various fields of science. The inclusion of these three databases improves the representativeness and accessibility of the data (Wan et al., 2024). We conducted searches at WoSCC, Pubmed, and Scopus, regardless of the language or type of literature, using the following search terms to retrieve data:

When searching at WOSCC, the search formula is as follows.

TS = (Gene Editing OR Genetic modification OR Genome editing OR DNA editing OR crispr_cas OR gene editing technology OR genome editing tool OR genetic engineering system OR base editing) AND (engineering system OR base editing) AND TS = (amyotrophic lateral sclerosis OR Lou Gehrig’s disease).

When searching PubMed, the search formula is as follows.

((Gene Editing) OR (Genetic modification) OR (Genome editing) OR (DNA editing) OR (crispr_cas) OR (gene editing technology) OR (genome editing tool) OR (genetic engineering system) OR (base editing)) AND ((amyotrophic lateral sclerosis) OR (Lou Gehrig’s disease)).

When searching Scopus, the search formula is as follows.

(ABS (Gene Editing) OR ABS (Genetic modification) OR ABS (Genome editing) OR ABS (DNA editing) OR ABS (crispr_cas) OR ABS (gene editing technology) OR ABS (genome editing tool) OR ABS (genetic engineering system) OR ABS (base editing) AND (ABS (amyotrophic lateral sclerosis) OR ABS (Lou Gehrig’s disease))).

**Figure 1:**
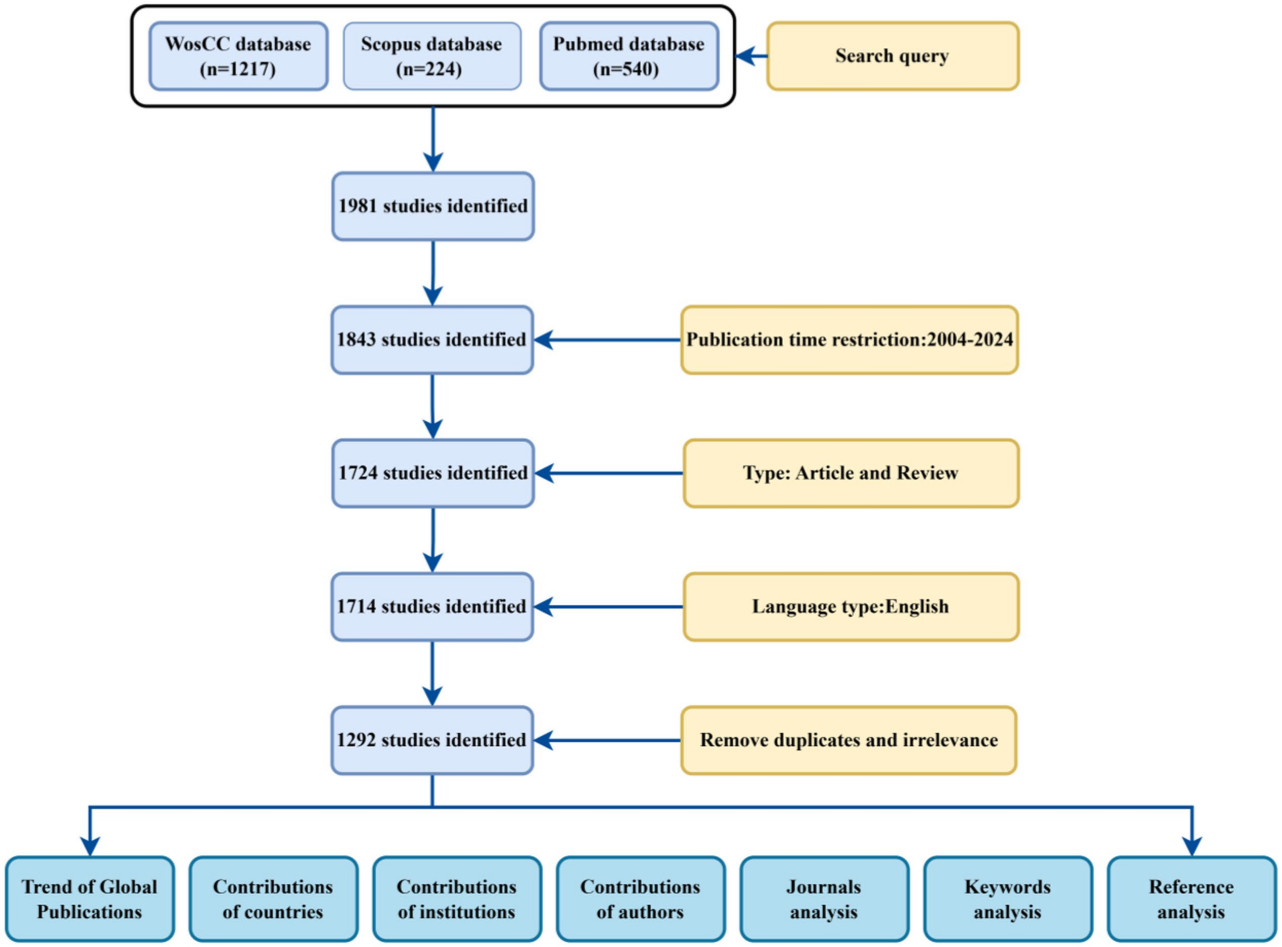
Flow chart of literature search and screening.

Retrieved data were collected on October 24, 2024 to avoid possible bias from daily updates. By restricting the publication period to 2004–2024 and choosing English as the language type, a total of 1981 documents were obtained; excluding out-of-time, conference abstracts, letters, and duplicates, as well as screening non-English language and missing data documents, a final total of 1,292 documents were included, which were visualized and analyzed, and global publication trends were mapped, country contributions were mapped, institutional contributions were mapped, author contributions were mapped, and journals were analyzed. A global publication trend map, a country contribution map, an author contribution map, a journal analysis map, a reference analysis map, a keyword analysis map and a keyword analysis map were drawn, as shown in Figure 1.

### 2.2 Data analysis

The 1,292 included documents were converted to Microsoft Office Excel 2019 and visualized and analyzed using the bibliometric software VOSviewer (v.1.6.19) developed by the Center for Scientific and Technological Research (CWTS) at Leiden University, The Netherlands (Ma et al., 2021) and the bibliometric software developed by Prof. Chiu-Mei Chen of Drexel University, U.S. (Li et al., 2023). CiteSpace (v.6.2 Advanced) for visualization and analysis.R has a unique advantage in being used to process large-scale literature for inclusion, while CiteSpace, with its unique algorithms, allows for in-depth and thorough analyses of institutions, authors, keywords, and citations (Chen, 2020). VOSviewer software, which analyzes more intuitive results compared to the above two software. By combining the advantages of the above three analytical tools, the map drawn from their visual analysis can predict the trend of the field, thus promoting in-depth research in the field (Synnestvedt et al., 2005) and playing an important role in the treatment of ALS patients.

In the process of using CiteSpace software, we exported the documents from WOS in the format of “Plain text file” and imported them into CiteSpace software, respectively. When we search in WOS, we can see an “Export” button, click it, we can see EndNote online, EndNote desktop, Plain text file and many other export formats. At this time, we need to click on the third line of the “Plain text file,” we need to visualize the file export.

The specific parameters of CiteSpace software are set as follows: network pruning method (Pathfinder), time slicing (1 year), *K* = 25, keyword clustering using LLR and LSI clustering algorithms. This setting, which comes from Prof. Chen’s design in the software, can balance the number of results and clarity, and show as many key words as possible, so as to achieve the most perfect state of presentation (Chen, 2020). Meanwhile, we also in the results presentation part, we remove the redundant information, only part of the visualized data images are retained conducive to logical interpretation of the results for the readers.

## 3 Results

### 3.1 Temporal distribution graph of literature

**Figure 2:**
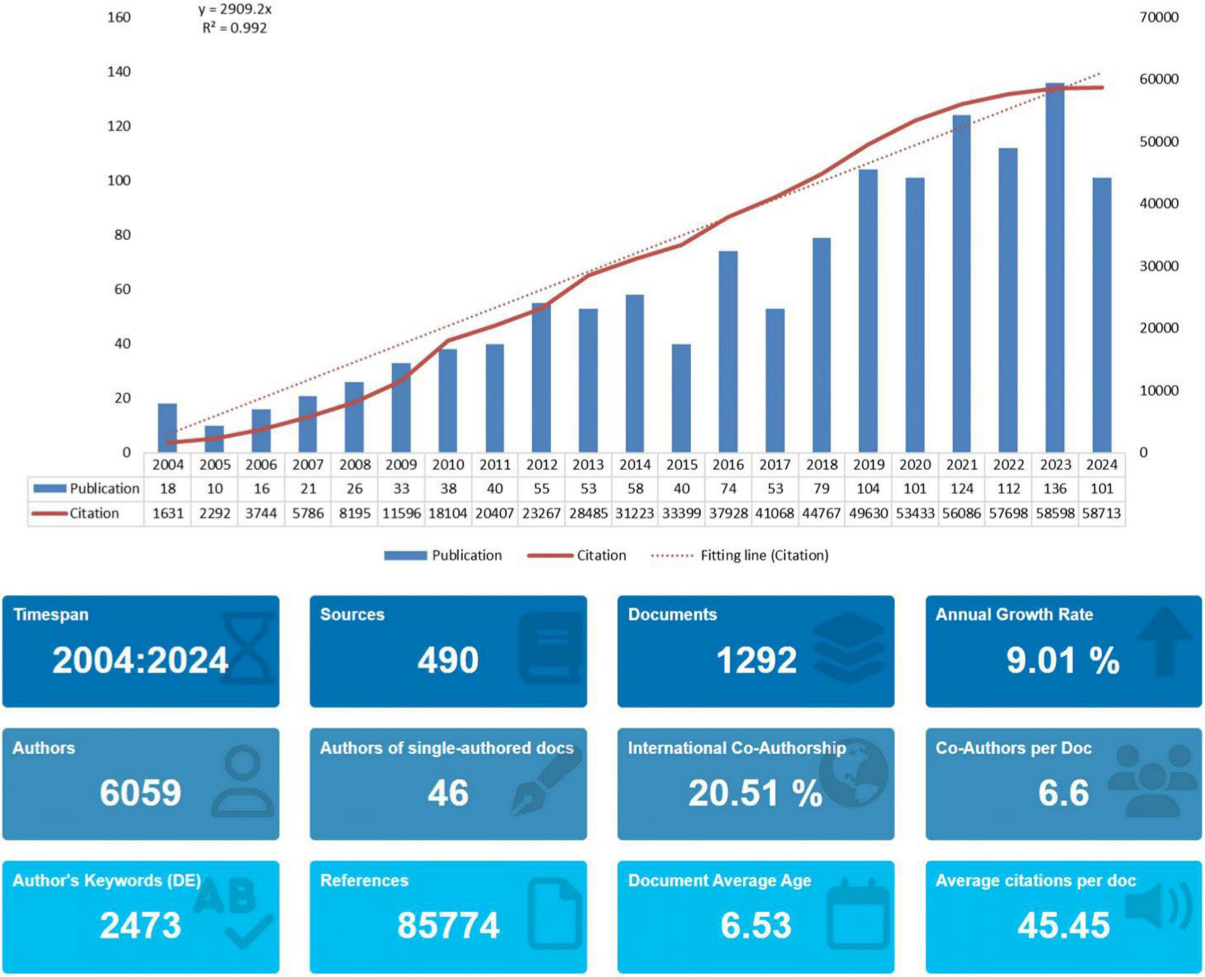
Temporal trends of publications (per year) and citation (cumulatively).

Changes in the number of papers over time reflect the level of research and trends in the field. As shown in Figure 2, several papers have been published in this field since 2004, indicating that the field has attracted the attention of researchers. Since 2005, the number of papers increased every year until 2012, and the number of publications in this field was relatively stable until the period between 2012 and 2014. Between 2015 and 2018, the number of papers published shows fluctuations, but the overall trend is upward. And then, in 2023, the number of papers published in this field peaked at 136. Meanwhile, in addition to the bar chart, Figure 2 also shows the fitted curve of the annual trend of paper publication. Although the number of papers has fluctuated in some years in the last 20 years, the correlation coefficient R^2^ is 0.992, which proves that the overall number of papers increases with the increase of years and is relatively stable, because the more R tends to be close to 1, which means that the model fit is more accurate. This shows that research related to gene editing and ALS has attracted much attention and is developing rapidly. We also projected the predicted straight line of article citations (*Y* = 2,909.2X), so it is reasonable to speculate that areas related to ALS and gene editing deserve to consistently receive academic attention in the future.

### 3.2 Trend of global publications and evolution of categories

**Figure 3:**
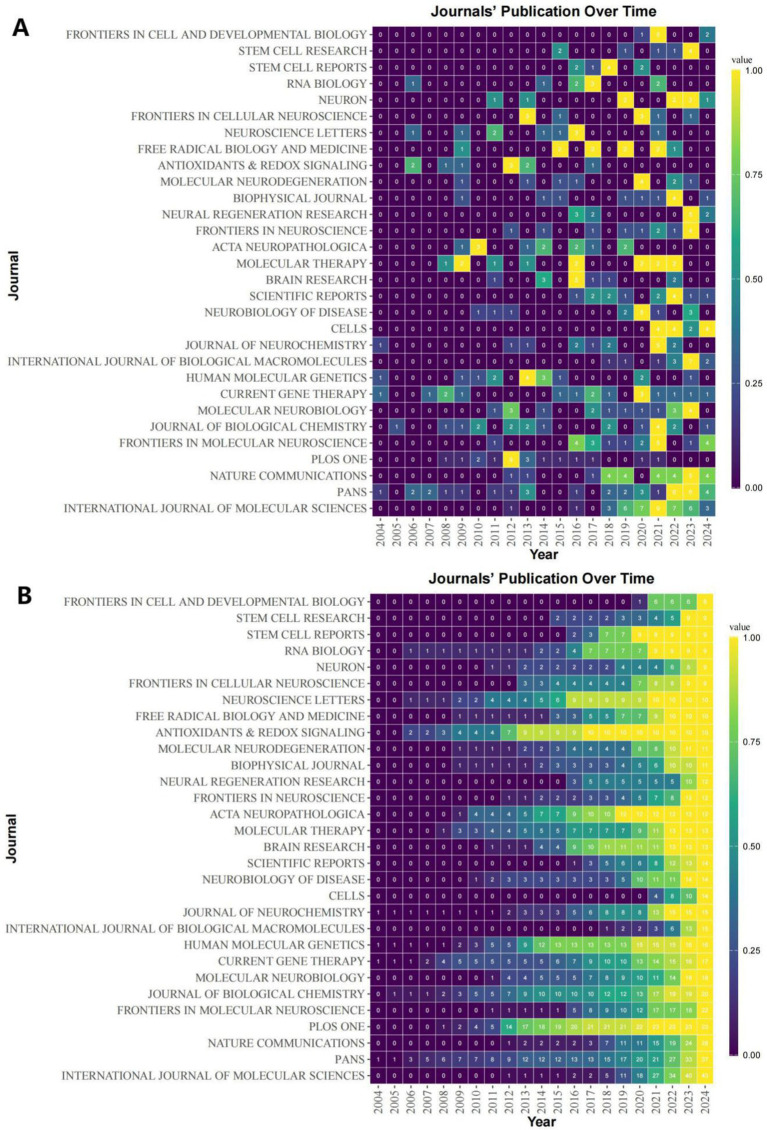
Heat map of journal publicationss. **(A)** Heat map of annual number of articles issued for 30 publications of interest. **(B)** Heat map of cumulative postings for 30 publications of interest.

As of October 24, 2024 (2004–2024), a total of 1,292 topic-matched papers were included in this study. Figure 3A shows the number of articles by year for each of the 30 journals that publish highly regarded papers in the field. We can visualize from the figure that the number of papers on ALS and gene editing has been increasing year by year, with CURRENT GENE THERAPY having had a publication in this field in 13 of the years 2004–2024. Figure 3B illustrates the cumulative number of publications in the journals, with PANS focusing on this area since 2004, reaching a cumulative total of 37 papers by 2024, with more sustained growth than many other journals. Table 1 shows the top 10 journals in terms of impact, with a higher h_index indicating a higher journal impact. Among these 30 journals, PROCEEDINGS OF THE NATIONAL ACADEMY OF SCIENCES OF THE UNITED STATES OF AMERICA (PNAS) has the highest h_index of 23, followed closely by INTERNATIONAL JOURNAL OF MOLECULAR SCIENCES and NATURE COMMUNICATIONS, with an h_index of 17. Through further searching of the literature, we found that PNAS has a number of highly cited seminal studies, two of which have had a profound impact (Wu et al., 2006) found that inflammatory NADPH oxidase may be a cause of amyotrophic lateral sclerosis, providing new insights into the pathology of amyotrophic lateral sclerosis (Gruzman et al., 2007) experimentally identified a common molecular signature in SOD1 in sporadic and familial amyotrophic lateral sclerosis, which provides new insights into the treatment of amyotrophic lateral sclerosis.

**Table 1:** Top 10 most influential journals in the field.

Source	h_index	g_index	m_index	TC	NP	PY_start
PROCEEDINGS OF THE NATIONAL ACADEMY OF SCIENCES OF THE UNITED STATES OF AMERICA	23	37	1.095238095	2,891	37	2004
INTERNATIONAL JOURNAL OF MOLECULAR SCIENCES	17	34	1.307692308	1,173	43	2012
NATURE COMMUNICATIONS	17	28	1.307692308	1,599	28	2012
PLOS ONE	16	23	0.941176471	1,228	23	2008
JOURNAL OF BIOLOGICAL CHEMISTRY	15	20	0.75	782	20	2005
FRONTIERS IN MOLECULAR NEUROSCIENCE	13	22	0.928571429	618	22	2011
HUMAN MOLECULAR GENETICS	13	16	0.619047619	1,163	16	2004
MOLECULAR THERAPY	13	13	0.764705882	1,388	13	2008
ACTA NEUROPATHOLOGICA	12	12	0.75	1,019	12	2009
JOURNAL OF NEUROCHEMISTRY	12	15	0.571428571	551	15	2004

### 3.3 Distribution of countries/regions

**Figure 4:**
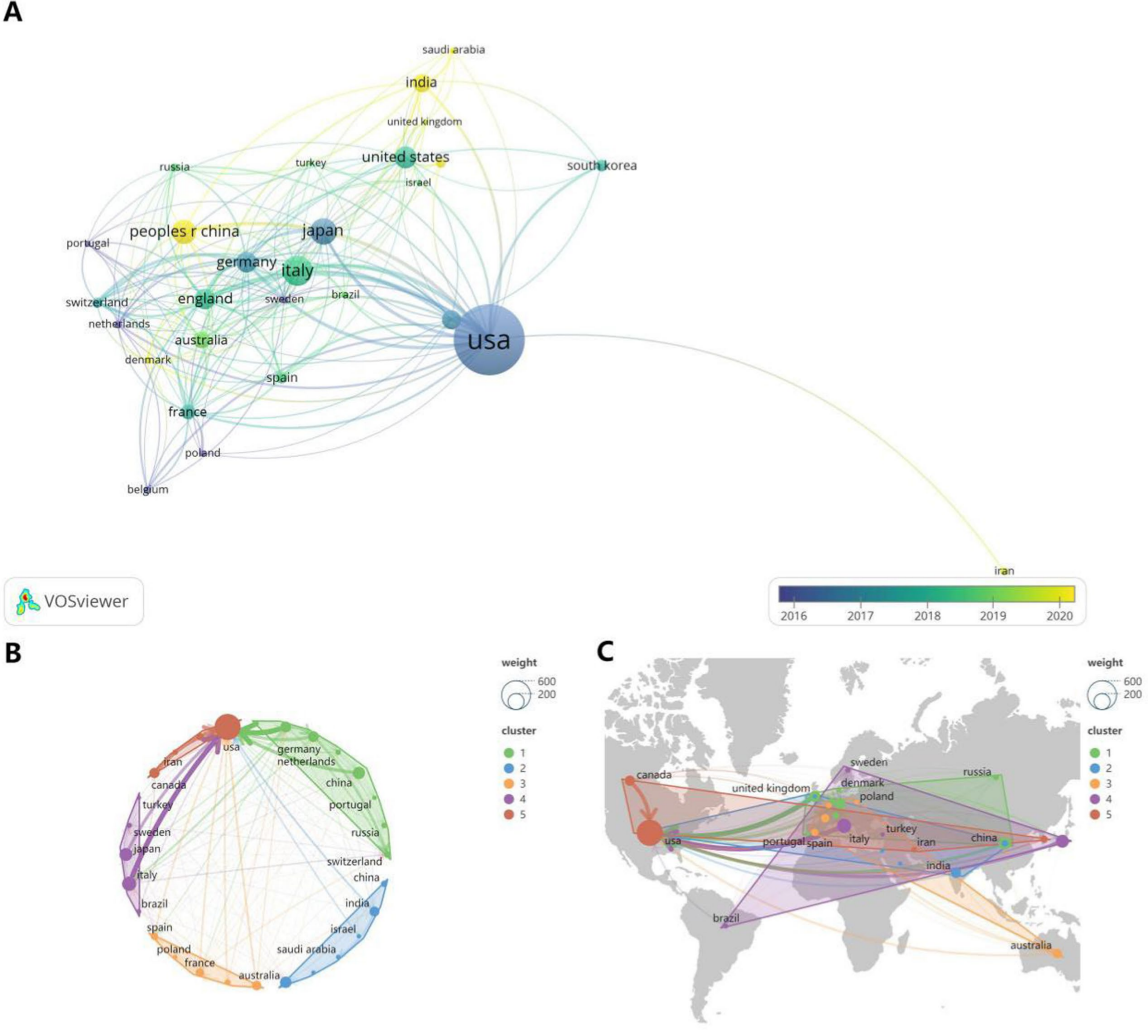
Country-area contribution diagram. **(A)** Timeline Chart for Country Prominence Publishing. **(B)** Clustered country cooperation circularity maps **(C)** World map of countries, regional cooperation.

In the last 20 years (2004–2024), a total of 1,292 WORKS on ALS and gene editing have been published worldwide. We selected the countries/regions with more than 5 publications for visualization and obtained the Timeline Chart for Country Prominence Publishing (Figure 4A). Among them, dark-colored countries such as USA, Japen, and Germany conducted research earlier, while yellow-colored countries such as China, and India started later. Figure 4B shows the Clustered country cooperation circularity maps, we can see that the green clusters have the most number of countries with 8 countries, while the red clusters have relatively few countries with only 4 countries. Figure 4C shows the World map of countries, regional cooperation, and we can see that there are more cross-continental cooperation in this field, which is more closely connected.

Based on the extraction of the number of articles published by each country, we can get the top 10 countries in this field (Table 2). Among them, the country with the highest number of articles is the United States with 343 articles, accounting for 26.54% of the total, followed by China with 79 articles, accounting for 6.11% of the total. Three of the top five countries in terms of the number of articles are from Asia, i.e., China, Japan and India. In addition to China, Japan ranked fourth and India ranked fifth with 71 and 48 articles respectively, accounting for 5.49 and 3.71% of the total number of articles.

**Table 2:** Top 10 countries in terms of number of publications.

Country	Articles	Articles %	SCP	MCP	MCP %
USA	343	26.54798762	278	65	18.95043732
China	79	6.114551084	71	8	10.12658228
Italy	78	6.037151703	63	15	19.23076923
Japan	71	5.495356037	59	12	16.90140845
India	48	3.715170279	34	14	29.16666667
Canada	46	3.560371517	34	12	26.08695652
Germany	43	3.328173375	30	13	30.23255814
United Kingdom	42	3.250773994	21	21	50
Australia	32	2.476780186	21	11	34.375
Korea	27	2.089783282	20	7	25.92592593

### 3.4 Contributions of institutions

**Figure 5:**
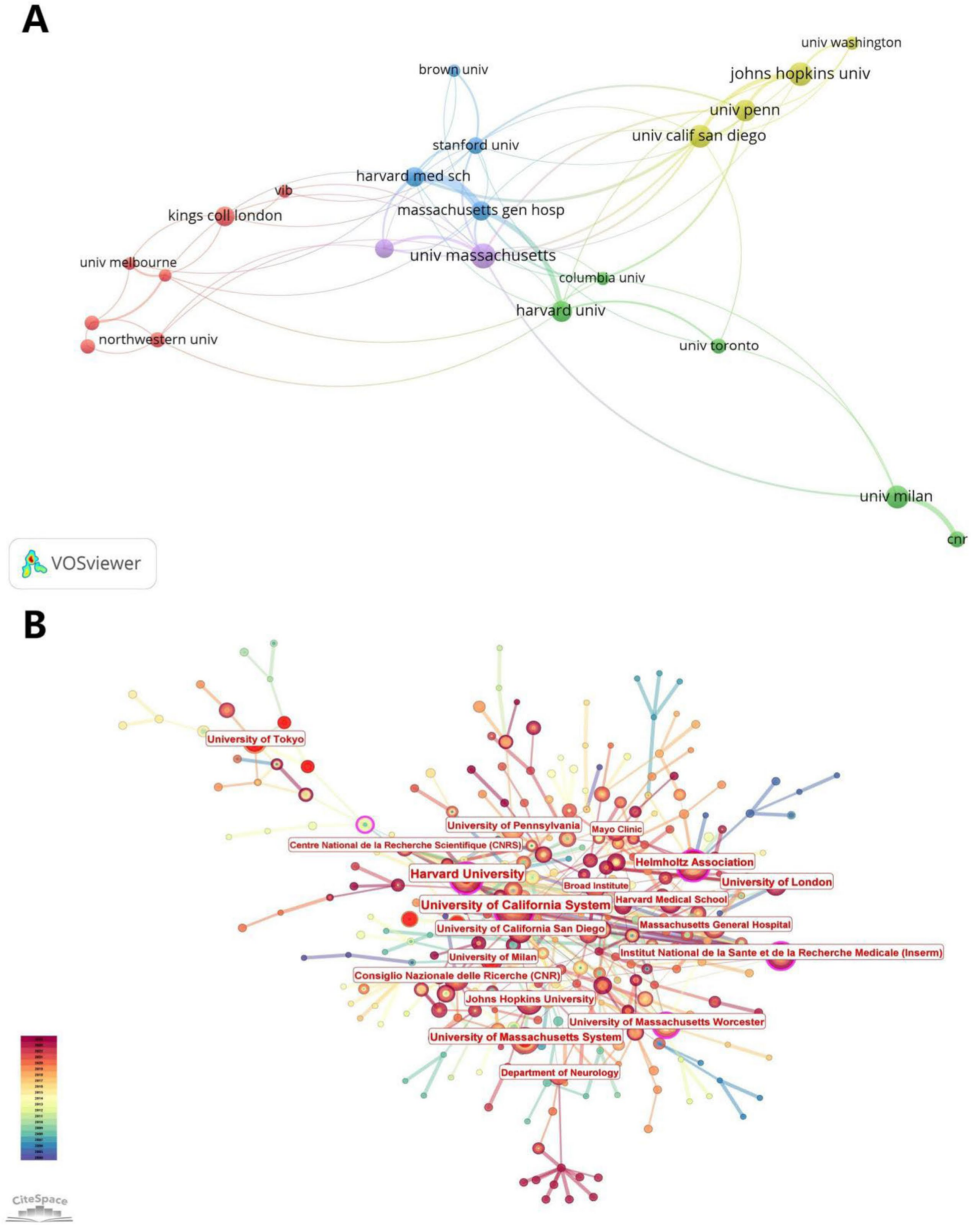
Contribution map of institutions and organizations. **(A)** Clustering map of institutions and organizations, with the same color indicating the same clustering. **(B)** Presentation map of contributing institutions and organizations, with larger gaps indicating greater centrality.

Figure 5 illustrates the visual analysis of the collaborative network of publishing organizations using VOSviewer and CiteSpace software. The results of the collaboration analysis using VOSviewer (Figure 5A) show that there are five clusters in the collaboration network, with the highest number of red clusters. The centers of each color cluster are harvard med sch, johns hopkins univ., univ. massachusetts, harvard univ., and kings coll london. According to the citation space analysis of the issuing institutions, University of California System, Harvard Medical Schoo, Institut National de la Sante et de la Recherche Medicale (Inserm), and University of London all appeared as core institutions in the co-occurrence map (Figure 5B), which demonstrates the study’s importance of the field. Table 1 shows the 20 institutions with the highest number of publications in this area, with univ. massachusetts and johns hopkins univ. publishing the highest number of papers (see Table 3).

**Table 3:** Top 20 organizations with publications in the field.

Id	Organization	Documents	Citations	Total link strength
1,671	Univ massachusetts	26	1855	19
830	Johns hopkins univ	24	1,246	12
1787	Univ tokyo	24	1,121	13
1,570	Univ calif san diego	23	1751	17
1,686	Univ milan	23	995	9
1724	Univ penn	21	1875	12
604	Harvard univ	20	4,762	12
388	Department of neurology	18	198	0
599	Harvard med sch	18	1,210	21
872	Kings coll london	18	921	5
975	Massachusetts gen hosp	18	2,144	23
999	Mayo clin	17	968	10
1,386	Stanford univ	15	1,289	15
270	Cnr	14	547	5
1,557	Univ british columbia	14	585	0
1,145	Northwestern univ	13	622	5
1790	Univ toronto	13	794	5
236	Chinese acad sci	12	456	2
1733	Univ queensland	12	346	5
279	Columbia univ	11	1,127	9

### 3.5 Authors and co-cited authors

**Figure 6:**
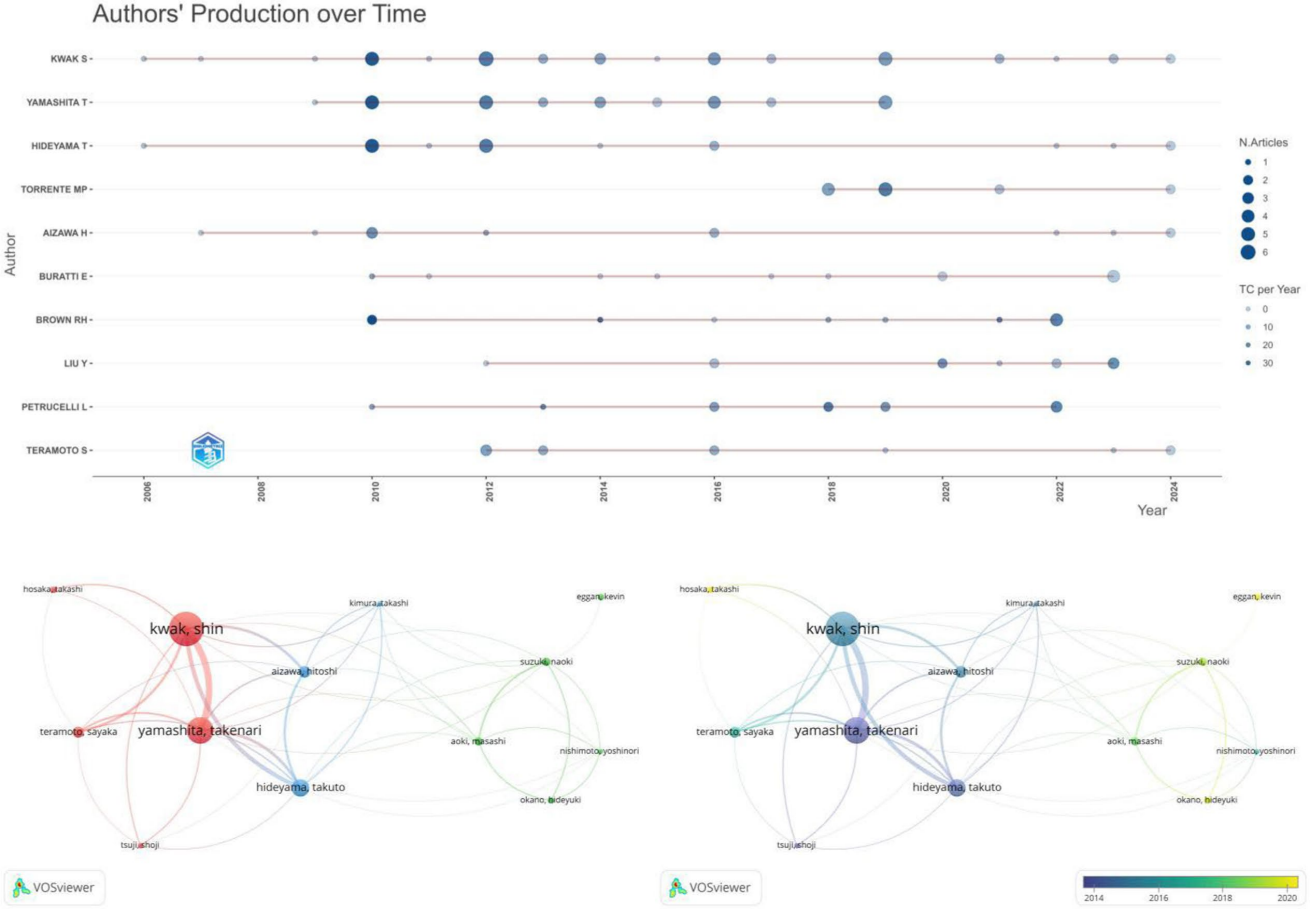
Map of authors and co-cited authors. **(A)** Timeline visualization of the top 10 authors of published literature in the field. **(B)** VOSviewer-based visualization of collaborations between authors **(C)** VOSviewer-based timeline visualization of clusters of author collaborations.

Figure 6A illustrates the top 10 authors in terms of the number of publications in the field, with KWAK S and YAMASHITA T being involved in the largest number of publications, KWAK S (39 papers published, number of citations: 367), YAMASHITA (29 papers published, number of citations: 331), followed by HIDEYAMA T, TORRENTE MP, and AIZAWA H. We further visualized the collaboration between authors of published papers to observe the degree of joint collaboration among the 13 scholars who published five or more papers. As shown in Figure 6B, authors collaborated with each other to form three clusters, but the different author clusters were not strongly connected to each other. In addition, we visualize the authors’ collaboration clusters in combination with the timeline to obtain (Figure 6C), from which it can be seen that authors with a large number of publications and citations have a relatively early start. The right part, on the other hand, started later but shows a growing trend.

### 3.6 Citation analysis

**Figure 7:**
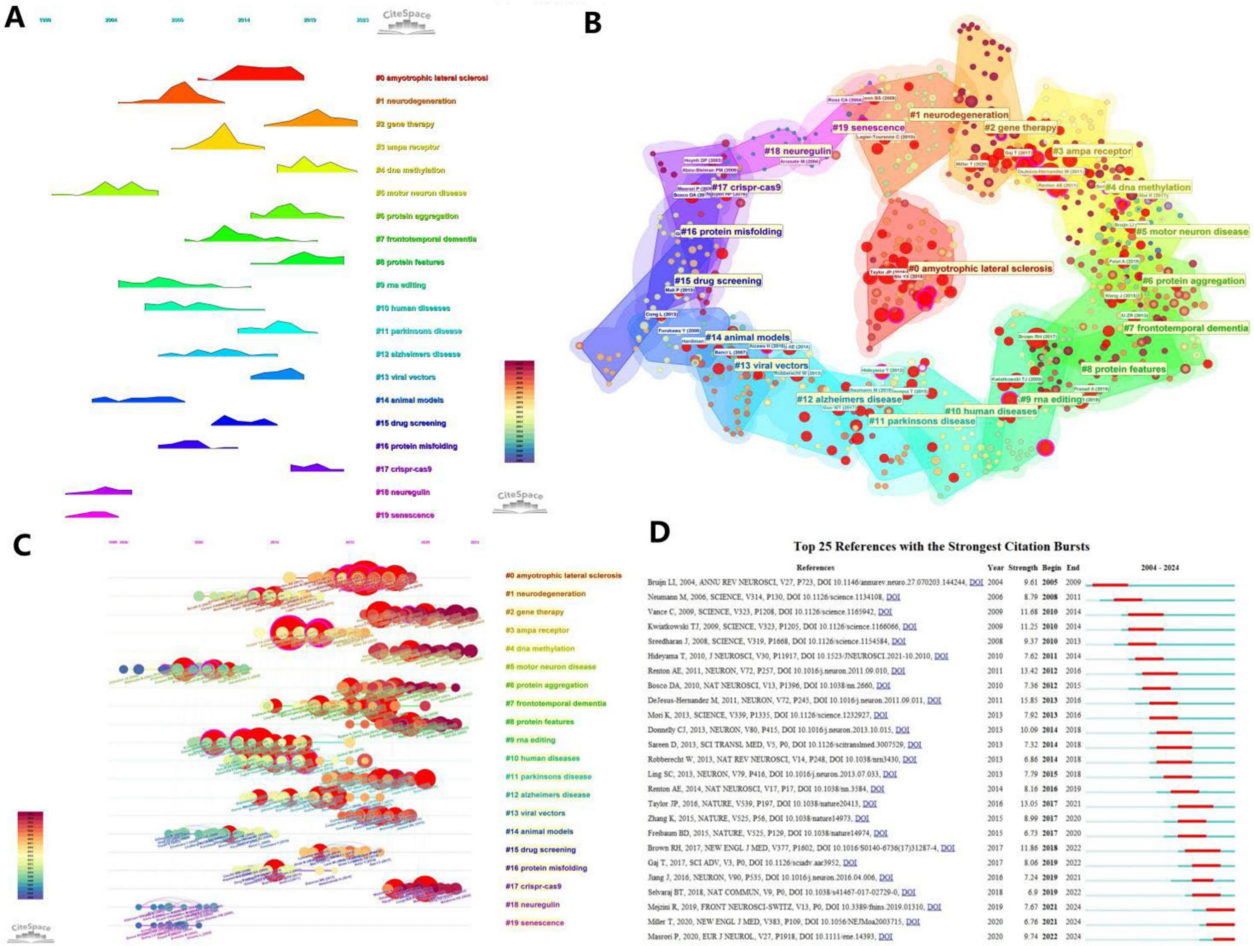
Visualization of references. **(A)** Timeline graph of the outbreak of cited literature; **(B)** Visualization of clustering of cited literature. **(C)** Visualization of keywords appearing in the cited literature combined with a timeline. **(D)** Bursts plot of cited literature, with the red portion representing duration.

Citation analysis allows for the analysis of the field that the article is corroborating, and often reveals the roots or cornerstones of a particular discipline, from which new disciplines often branch out. Figure 7A presents a timeline of the concentration of citations for each cluster topic, and Figure 7B visualizes the number of major articles included in each cluster, with the red circles representing the core literature. Figure 7C builds on Figure 7A by showing which literature is specifically cited centrally. Figure 7D shows the top 25 most cited references of all literature with high reference value.

The keyword frequencies of the cited literature in each year were further analyzed, and the 25 literature with the strongest outburst of citations were extracted (Figure 7D). The results show that among these 25 documents, the journals with the most cited source documents are SCIENCE and NEURON with 5 occurrences each, followed by NATURE with 3 occurrences. The most cited literature was Bruijn et al. (2004), a systematic review of the mechanisms of motor neuron degeneration in ALS, followed by Neumann et al. (2006) ubiquitinated TDP-43 in frontotemporal lobar degenerative diseases and amyotrophic lateral sclerosis, and then by Vance et al. (2009), a study that examined the effects of ubiquitination on motor neuron degeneration by localization analysis of mutant proteins in a British lineage with familial (ALS) type 6, identifying missense mutations in the gene encoding fusion sarcolemmal protein (FUS), revealing the underlying mechanism of ALS.

Among the recent explosion of cited studies are equally compelling papers. For example, (Gaj et al., 2017) successfully reduced muscle atrophy in ALS model mice using the CRISPR-Cas9 system to specifically disrupt mutant SOD1 gene expression in their study. It provides a precise and controlled method to modify disease-causing genes. This approach has broad implications for the field, as it not only provides a valuable tool for studying disease mechanisms, but also paves the way for the development of personalized medicine and targeted therapies. Another example, (Selvaraj et al., 2018) found, through a combination of RNA sequencing and electrophysiological studies, that repeated amplification of the C9ORF72 gene resulted in increased expression of the GluA1 AMPA receptor subunit, making motor neurons more sensitive to Ca2 + −permeable AMPA receptor-mediated excitotoxicity, a finding that, from the point of view of neuronal cellular signaling pathways reveals new potential triggers for ALS production, which is important for understanding disease progression and developing neuroprotective strategies. It can be seen that gene therapy is currently a more popular research direction, both in the exploration of ALS pathogenesis and in the construction and validation of therapeutic methods.

### 3.7 Keyword analysis

**Figure 8:**
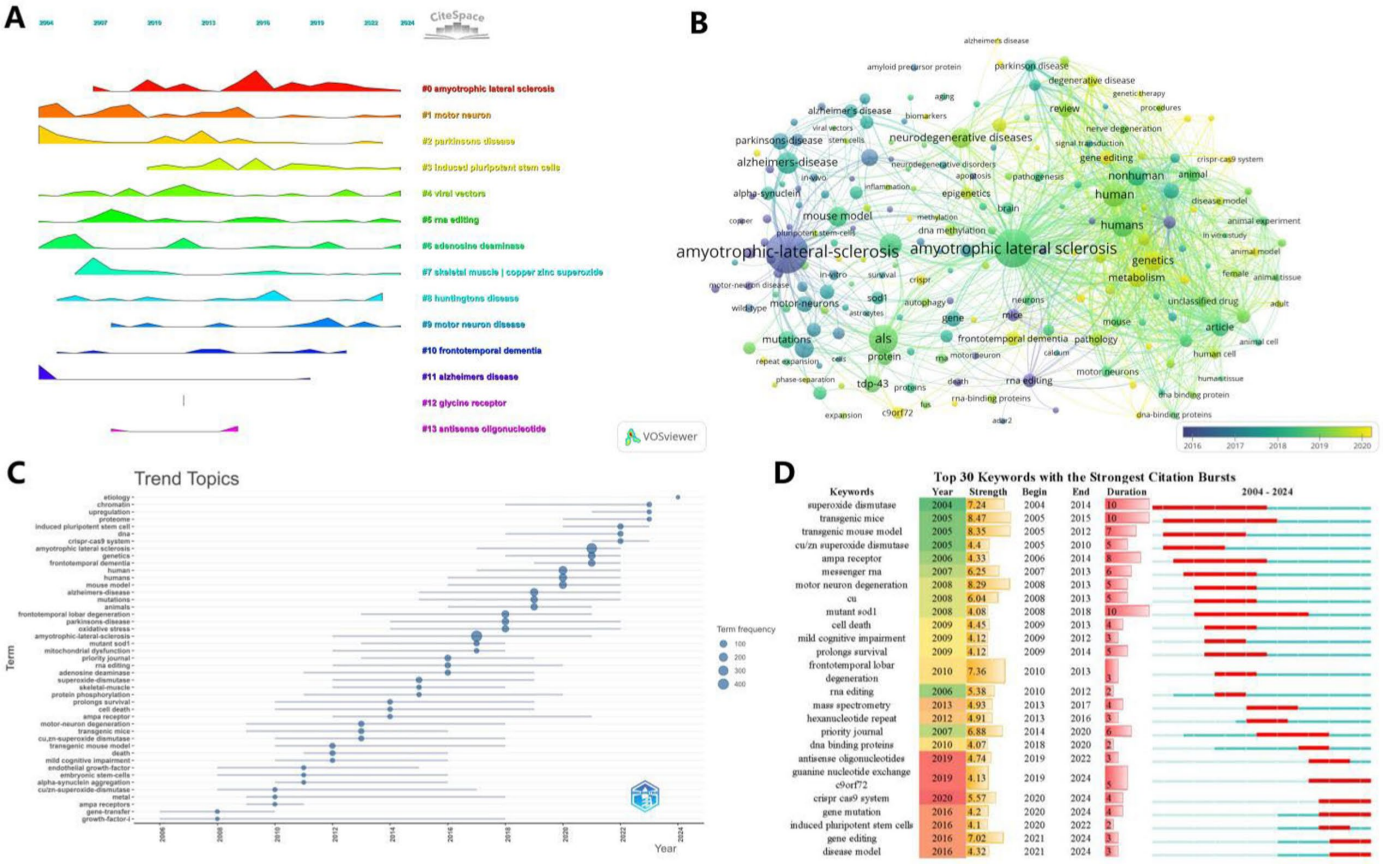
Visualization of keywords or subject terms. **(A)** Visualization of keyword clustering combined with timeline. **(B)** Timeline visualization of keyword co-occurrence. Keyword nodes with earlier occurrences are colored closer to purple, and keyword nodes with more recent occurrences are colored closer to yellow. **(C)** Timeline visualization of the first 45 subject terms. **(D)** Keyword Bursts graph, with the red portion representing duration (Figure 9).

Keyword analysis demonstrates trends and hotspots in a particular research area. At the time of keyword analysis, all 1,292 matching documents were included. Through citespace’s clustering analysis of keywords, we can get the trend of keyword clustering over time (Figure 8A). At the same time, we can get the results of (Figure 8D), which shows the 30 most prominent keywords among them as well as other more specific analyses. Further, using VOSviewer it is possible to obtain the specific time of the outbreak of individual keywords, which is presented in Figure 8B. In order to get a macro view of the themes developing in the field as a whole, we used R software to analyze the subject terms of the field and show the duration of their development, which is presented in Figure 8C as the length of the keywords’ bolded lines.

**Figure 9:**
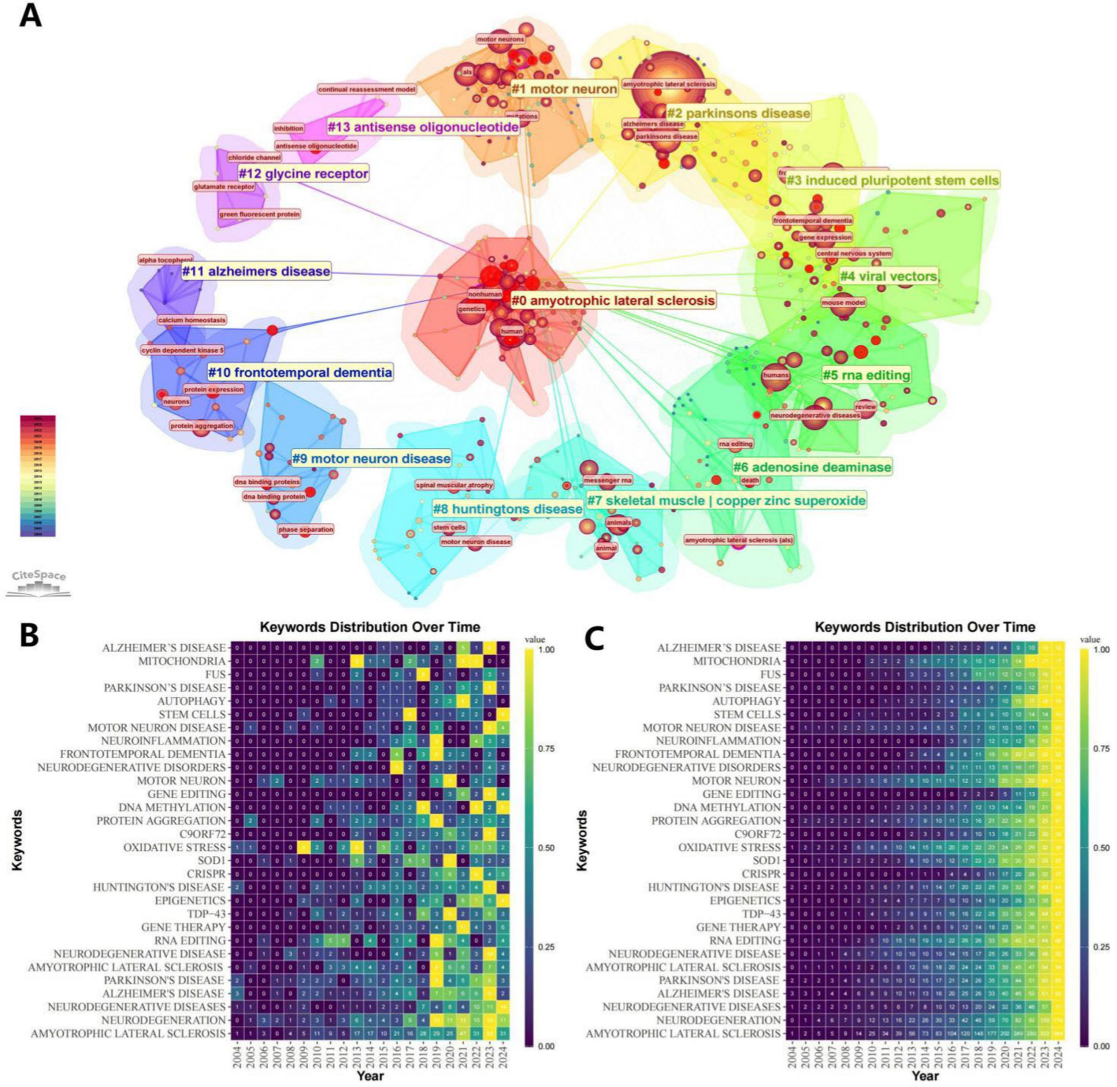
Keyword Visualization Map. **(A)** Keyword clustering map. The same clusters are located under the same color block, and the outer pink circles represent keywords with strong centrality. **(B)** Keyword distribution frequency heatmap. **(C)** Keyword cumulative frequency heatmap.

The information provided in Figure 9 is more specific. Figure 9A shows the more detailed keywords under each clustered theme. Figures 9B,C, a representative keyword is meticulously analyzed by R software, recording the value it contributes per year and the cumulative value it contributes, respectively.

## 4 Discussion

### 4.1 General information

This review was written after visual analysis of the included literature using CiteSpace, VOSviewer and R software to analyze cited articles, cited authors, keyword contributions, cited journals and emerging keywords. Since 2004, research in the fields of ALS (amyotrophic lateral sclerosis) and gene editing has been on an overall upward trend with an increasing number of citations. Based on the fitted straight line of the publication year trend in Figure 2, the number of articles is increasing year by year and there is plenty of room for future research. Further statistical analysis and mapping of publishing organizations and countries/regions can identify countries/regions and publishing organizations that are conducting more research in the field with greater impact and closer collaboration. Among them, the United States has conducted the most research in the field. We also find that Germany, China and Russia collaborate relatively more closely in the largest clusters. Among the research organizations, univ. massachusetts in the United States and kings coll london in the United Kingdom are influential with large contributions. In the author analysis section, KWAK S, YAMASHITA T and HIDEYAMA T made a large contribution and are advanced scholars in this field.

In addition to the above basic information, journal analysis, citation analysis and keyword analysis are particularly important. h_index is generally used as an important measure of a journal, reflecting the quality of the works published by the journal. Works in PNAS are of high scholarly value due to their groundbreaking discoveries. In the citation analysis, we found that the directions #2 (Gene Therapy), #4 (DNA Methylation), #6 (Protein Aggregation), and #13 (Viral Vectors) are the more cited areas in the last decade, and are expected to be the basis of the emerging discipline of gene editing in the field of tachyzoites in the future as well; Meanwhile, traditional research directions such as #3 (AMPA Receptor), #14 (Animal Models), and #16 (Protein Misfolding) cannot be ignored. Keyword analysis was particularly important, and we found key compounds such as superoxide dismutase, mutant sod1, and AMPA receptor among the most prominent 30 keywords, as well as molecular signals such as hexanucleotide repeat, dna binding proteins, crispr cas9 system. This has enlightened us as to the angles from which we should follow up with new research.

### 4.2 Als gene-related etiology

ALS (amyotrophic lateral sclerosis) research has entered a phase of in-depth exploration at the genetic level, and mutations in several key genes are closely related to the pathogenesis of ALS.

Mutations in the SOD1 gene were one of the first genes found to be associated with ALS, especially in a high proportion of familial ALS cases. Mutations in SOD1 encode a superoxide dismutase 1 protein that loses its normal antioxidant function, generating oxidative stress that damages motor neurons (Kitaoka et al., 2023). In addition, aggregation of SOD1 protein leads to mitochondrial dysfunction and disruption of intracellular calcium homeostasis, which further exacerbates degenerative changes in neurons (Abati et al., 2020).

Pathological accumulation of the TDP-43 protein due to mutations in the TARDBP gene is another hallmark feature of ALS.TDP-43 not only participates in RNA processing and transport as an RNA-binding protein but its misfolding and aggregation trigger disruption of protein homeostasis in ALS patients (Kurashige et al., 2022). It has been found that TDP-43 aggregation is mainly concentrated in the cytoplasm of neurons, which affects the normal function of several signaling pathways, including mRNA metabolism and autophagy pathways (Oiwa et al., 2023).

Mutations in the C9orf72 gene are strongly associated with hexanucleotide repeat expansions, and this mutation is particularly common in patients with familial ALS (Zhang et al., 2018). Repeat expansion produces toxic RNA and protein aggregates that have toxic effects on neurons, triggering intracellular stress responses and ribosomal dysfunction (Balendra et al., 2016).

Mutations in the KIF5A gene are thought to be associated with abnormal long-range axonal transport function in ALS, which results in motor neurons not being able to properly transport required nutrients, thereby accelerating neuronal degeneration (Gu et al., 2019). Studies have shown that restoring the normal function of KIF5A may help to slow down the progression of ALS (Nakano et al., 2022).

Other genes such as FUS, OPTN, and TBK1 also play important roles in the pathogenesis of ALS (Parakh et al., 2021; Oliveira et al., 2021; Rajpurohit et al., 2020). Mutations in these genes involve abnormalities in several aspects of intracellular autophagy function, stress response, and mitochondrial function, and the cumulative effect of these mechanisms further accelerates the course of ALS.

### 4.3 Application of gene editing in ALS

Gene editing technologies have shown great potential for application in the field of amyotrophic lateral sclerosis (ALS) treatment in recent years, especially in the CRISPR-Cas system, antisense oligonucleotide (ASO), and RNA editing technologies, where significant progress has been made. These technologies have opened up new avenues for personalized treatment of ALS, capable of targeting specific genetic variants to modify or block the disease process.

CRISPR-Cas technology, as an efficient gene editing tool, has been initially applied to modeling studies of ALS. Using CRISPR-Cas9 technology, researchers were able to correct disease-causing gene mutations in patient-derived induced pluripotent stem cells (iPSCs) to generate cellular models of ALS for disease mechanism studies and drug screening. For example, Wang et al. corrected the SOD1 gene mutation in iPSCs from ALS patients using CRISPR-Cas9 technology and successfully restored the normal function of neurons (Wang et al., 2017). In addition, CRISPR technology has been used to construct ALS models with specific gene mutations, revealing the pathogenic mechanism of the SOD1 gene (Kruminis-Kaszkiel et al., 2018).

In terms of antisense oligonucleotide (ASO) technology, which reduces the production of disease-causing proteins by binding to the target mRNA and either inhibiting its translation or facilitating its degradation, ASO technology has shown significant efficacy in SOD1 mutation-associated ALS (Lopez et al., 2022). For example, Tofersen, an antisense oligonucleotide targeting SOD1 mRNA, has been shown in preliminary data to be able to reduce SOD1 protein levels and slow disease progression (Miller et al., 2020). In addition, ASO technology has also been used to target C9orf72 repeat expansions and FUS mutations, showing good therapeutic promise (Korobeynikov et al., 2022).

RNA editing technology can affect protein function by directly modifying mRNA sequences, either by altering the expression of specific genes or by correcting mutations (Gao et al., 2021a,b). In early studies of ALS, premature death in mice with defective GluR2 mRNA editing was caused by neuronal death2 but could be rescued by restoring RNA editing function (Higuchi et al., 2000). On this basis, Hideyama’s team found that ADAR2 is the key enzyme responsible for RNA editing, especially for the editing of the Q/R site (glutamine/arginine site) of the AMPA receptor subunit GluA2, and that RNA editing of this site is essential for preventing the excessive influx of Ca2+ ions and nerve cell excitotoxicity in nerve cells (Hideyama et al., 2012). These studies on RNA editing technology have provided new ideas for the treatment of ALS, especially for mutations that cannot be corrected by conventional gene editing techniques.

### 4.4 How gene editing can be practically implemented for ALS patients

The application of gene editing in the clinic can be broadly categorized into *ex vivo* and *in vivo* applications. *In vitro* editing of genes for the production of antibodies (Li et al., 2020) or as an assay (Huang et al., 2023) is the most common and widespread application. In addition, the *in vivo* use of genes for direct delivery of therapeutics has also gained momentum, with the milestone being the first FDA approval of LNP-delivered *in vivo* CRISPR gene editing for human clinical trials (Ou et al., 2020).

Gene editing technology provides a new therapeutic pathway for ALS patients, and we can consider clinical application in two directions: off-target editing and *in vivo* direct delivery of gene editing. However, it is worth noting that off-target effects and immune responses must be strictly controlled during the implementation process to ensure the safety of the treatment. In addition, the clinical application of gene editing technology needs to follow strict ethical and legal standards to address potential social and ethical challenges.

### 4.5 The shortcomings of gene editing itself and its applications in ALS

CRISPR-Cas9 and antisense oligonucleotides (ASOs) have shown remarkable potential as gene editing technologies for the treatment of amyotrophic lateral sclerosis (ALS), but there are still challenges in terms of delivery systems, uncertainty of efficacy, and ethical issues on which researchers need to explore solutions.

Firstly, limitations in delivery systems are one of the major challenges facing CRISPR-Cas9 and ASO in ALS therapy. CRISPR-Cas9 relies on viral vectors (e.g., adeno-associated virus, AAV) or nanoparticles to deliver the editing tools into neurons, but these delivery systems often suffer from cell-type selectivity and limited distribution *in vivo*, especially in neurological structures such as the brain and the spinal cord, among other neurological structures. In addition, the immunogenicity of AAV vectors may lead to an immune response in patients, which can affect the safety and efficacy of the treatment (Mout et al., 2017). To avoid these problems, researchers are developing non-viral vectors (Lin et al., 2018), as well as novel AAV serotypes that are more targeted (Nyberg et al., 2023) to improve the efficiency and safety of CRISPR-Cas9 delivery in the nervous system of ALS patients. For ASO, non-viral-mediated direct injection strategies, have been used to enhance its delivery in the CNS, but optimizing the dose and frequency of delivery still requires further investigation (Edinoff et al., 2021).

Secondly, the uncertainty of efficacy is also a big limitation. CRISPR-Cas9, as a gene editing tool, can produce permanent genetic modifications; however, there is still a lack of data to support the long-term efficacy and safety of genetic modification, and unpredictable accidents still occur after CRISPR-Cas9 editing, such as off-targeting (Guo et al., 2023), unintended genetic changes (Pyenson et al., 2017), etc., affecting the health and stability of normal cells. In ASO therapy, ASOs need to be administered regularly to maintain the effect, which increases patient burden and may generate cumulative toxicity (Agarwal et al., 2022). These problems urgently need to be addressed by scientists by proposing new programs.

Ethical issues cannot be ignored aspects. Gene editing technology of CRISPR-Cas9 may affect the patient’s germ cells, which may lead to ethical controversies, especially in the editing of human embryos or germ cells (Coller, 2019). To minimize ethical controversies, research should be strictly limited to somatic cell editing, and strict off-target detection and assessment methods should be used to ensure the accuracy and safety of editing. For ASO, although it does not involve permanent genetic modification, its long-term use and possible side effects also need to be fully considered ethically.

### 4.6 Limitations

In this paper, we conducted a bibliometric analysis of gene editing and its application in amyotrophic lateral sclerosis (ALS) research with some limitations, which are listed below as the 4 main limitations of this study:

1. Database selection limitation: only three major databases, Web of Science Core Collection, PubMed and Scopus, were included in this study for the literature search. This may have limited our ability to access a wider range of literature, as other databases and gray literature may contain additional studies related to ALS and gene editing.
2. Timeframe limitation: we limited our analysis to literature published between 2004 and 2024, which may have excluded important studies outside of this timeframe, particularly those earlier studies that have historical implications for the current study.
3. Language restriction: this study included literature in English only, which may have excluded important research in other languages, especially those conducted in non-English speaking countries.
4. Literature type restriction: our search strategy may have excluded informal publications such as conference abstracts, reports, and dissertations that may contain valuable preliminary data and findings related to ALS and gene editing.

## 5 Conclusion

This paper analyzes the literature on gene editing in ALS research from 2004 to 2024 and finds a steady increase in the number of papers and citations in this field, which suggests that the field is maturing and interest in it is growing. The United States has contributed the most, followed by Germany, China and Russia with relatively closer collaboration. Univ. massachusetts and kings coll london are among the important institutions that have played a significant role in advancing the field. An analysis of keywords and references highlights the importance of #2 (Gene Therapy), #4 (DNA Methylation), #6 (Protein Aggregation), and #13 (Viral Vectors) as a focus of research, which is shifting towards understanding the biological mechanisms of ALS.Cu/Zn The role of SOD1 and AMPA receptors in the pathogenesis of ALS has emerged as an important area of research. Misfolding and aggregation of SOD1 and regulation of AMPA receptors have shown promise as potential therapeutic targets. Motor neuron degeneration involving TDP-43, FUS, and SOD1 remains a central theme in ALS research.

Despite the progress made, this bibliometric study recognizes limitations, such as reliance on the Web of Science Core Collection, PubMed and Scopus, and the exclusion of early-access literature prior to 2004. Future research should continue to explore the molecular mechanisms of ALS, with a focus on gene editing technologies, in order to develop effective treatments. The findings of this study provide a comprehensive overview of the current status, trends, and potential directions of ALS research, and provide guidance for researchers and clinicians seeking to improve diagnostic and therapeutic strategies for this devastating disease.

## Data Availability

The original contributions presented in the study are included in the article/supplementary material, further inquiries can be directed to the corresponding authors.
